# From Monoamines to Systems Psychiatry: Rewiring Depression Science and Care (1960s–2025)

**DOI:** 10.3390/biomedicines14010035

**Published:** 2025-12-23

**Authors:** Masaru Tanaka

**Affiliations:** Danube Neuroscience Research Laboratory, HUN-REN-SZTE Neuroscience Research Group, Hungarian Research Network, University of Szeged (HUN-REN-SZTE), Tisza Lajos krt. 113, H-6725 Szeged, Hungary; tanaka.masaru.1@med.u-szeged.hu; Tel.: +36-62-342-847

**Keywords:** major depressive disorder (MDD), neurocircuitry, synaptic plasticity, neuroimmune interactions, metabolic syndrome, treatment-resistant depression (TRD), neuromodulation, biomarkers, kynurenine, precision medicine

## Abstract

Major depressive disorder (MDD) was long framed as a single clinical entity arising from a linear stress–monoamine–hypothalamic–pituitary–adrenal (HPA) axis cascade. This view was shaped by forced swim and learned helplessness tests in animals and by short-term symptom-based trials using scales such as the Hamilton Depression Rating Scale (HAM-D) and the Montgomery–Åsberg Depression Rating Scale (MADRS). This “unitary cascade” view has been dismantled by advances in neuroimaging, immune–metabolic profiling, sleep phenotyping, and plasticity markers, which reveal divergent circuit-level, inflammatory, and chronobiological patterns across anxiety-linked, pain-burdened, and cognitively weighted depressive presentations, all characterized by high rates of non-response and relapse. Translationally, face-valid rodent assays that equated immobility with despair have yielded limited bedside benefit, whereas cross-species bridges—electroencephalography (EEG) motifs, rapid eye movement (REM) architecture, effort-based reward tasks, and inflammatory/metabolic panels—are beginning to provide mechanistically grounded, clinically actionable readouts. In current practice, depression care is shifting toward systems psychiatry: inflammation-high and metabolic-high archetypes, anhedonia- and circadian-dominant subgroups, formal treatment-resistant depression (TRD) staging, connectivity-guided neuromodulation, esketamine, selected pharmacogenomic panels, and early digital phenotyping, as endpoints broaden to functioning and durability. A central gap is that heterogeneity is acknowledged but rarely built into trial design or implementation. This perspective advances a plasticity-centered systems psychiatry in which a testable prediction is that manipulating defined prefrontal–striatal and prefrontal–limbic circuits in sex-balanced, chronic-stress models will reproduce human network-defined biotypes and treatment response, and proposes hybrid effectiveness–implementation platforms that embed immune–metabolic and sleep panels, circuit-sensitive tasks, and digital monitoring under a shared, preregistered data standard.

## 1. Framing the Journey—Prompts

**Figure 1:**
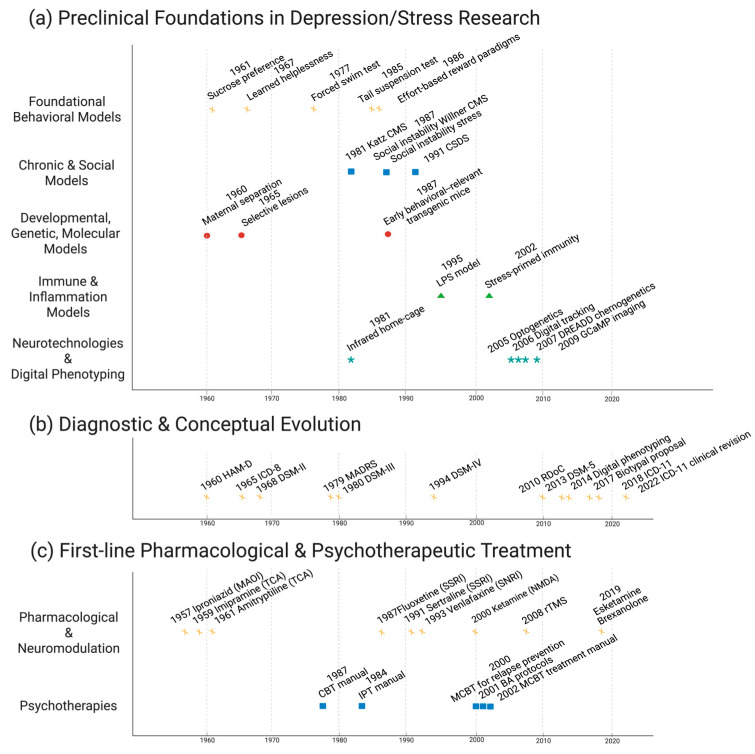
Translational timeline linking preclinical models, diagnostic frameworks, and first-line treatments in depression. The schematic illustrates the historical progression from early stress- and monoamine-based preclinical models, through key diagnostic milestones, to successive generations of pharmacological, psychotherapeutic, neuromodulatory, and digital interventions. The timeline highlights periods in which advances in experimental models, clinical nosology, and frontline care evolved in parallel, as well as phases in which these domains diverged, helping to contextualize current efforts to reconnect mechanism, diagnosis, and treatment within a systems psychiatry framework. Created in BioRender. Tanaka, M. (2025) https://BioRender.com/3omdcgb (accessed on 1 December 2025). BA, behavioral activation; CBT, cognitive behavioral therapy; CMS, chronic mild stress; CSDS, chronic social defeat stress; DREADD, designer receptors exclusively activated by designer drugs; DSM, Diagnostic and Statistical Manual of Mental Disorders; GCaMP, green fluorescent protein–calmodulin–M13 peptide; HAM-D, Hamilton Depression Rating Scale; ICD, International Classification of Diseases; IPT, interpersonal psychotherapy; LPS, lipopolysaccharide; MADRS, Montgomery–Åsberg Depression Rating Scale; MAOI, monoamine oxidase inhibitor; MBCT, mindfulness-based cognitive therapy; NMDA, N-methyl-D-aspartate; RDoC, Research Domain Criteria; rTMS, repetitive transcranial magnetic stimulation; SNRI, serotonin–norepinephrine reuptake inhibitor; SSRI, selective serotonin reuptake inhibitor; TCA, tricyclic antidepressant.

Major depressive disorder (MDD) has long been framed through a monoamine-centric lens, a narrative that catalyzed pharmacotherapy yet left substantial non-response and relapse [1]. Historical reassessments of the monoamine hypothesis and contemporary work on clinical, neurobiological, and treatment advances now converge on a simple conclusion: incremental refinements of the same model are no longer sufficient [2,3]. In an era of high-dimensional neuroimaging, immune profiling, and digital phenotyping, revisiting the trajectory from 1960s monoamine theories to contemporary systems psychiatry is therefore not merely historical, but strategically necessary [4,5] (Figure 1).

This perspective views MDD as a nexus condition with dense crosstalk with anxiety, chronic pain, and dementia, where shared and distinct pathways blur categorical boundaries [6]. Large-scale cohort and imaging studies reveal common yet dissociable alterations in prefrontal and limbic circuitry across depression, anxiety, post-traumatic stress disorder, and pain, while mechanistic work highlights bidirectional links with cardiometabolic and neurodegenerative disease [7,8,9]. Within this landscape, anxious, pain-enriched, and cognitively loaded depression exemplify how comorbidity patterns reshape prognosis, treatment response, and trial outcomes [10,11].

Our central thread traces the shift from neurotransmitter-centered framework toward an integrated immune, metabolic, and circuit-based framework that can anchor precision care [12]. This perspective synthesizes evidence on central–peripheral immune interactions, network-level biotypes, and model-driven subtypes of brain organization, and outlines how these advances should inform next-generation therapeutics and trial design [13,14,15]. Rather than providing another unitary model of depression, this perspective advances frameworks that treat heterogeneity as signal, not noise, enabling stratified, personalized interventions across intertwined spectra of mood, anxiety, pain, and cognitive decline [16] (Box 1).

Box 1 **Key Terms.**

A *biomarker* is an objective biological signal that indexes state, risk, or treatment response. An *endophenotype* reflects a heritable, mechanistic intermediate—closer to circuitry than symptoms. *Anhedonia* captures reduced reward sensitivity or effort, a core transdiagnostic deficit. *Treatment-resistant* denotes limited response after adequate therapeutic trials. *Rapid-acting* refers to interventions producing meaningful change within hours to days. A *network target* is a circuit-defined node guiding drug or device precision. A *digital endpoint* leverages passive or task-based sensors to quantify behavior in real time.

## 2. Early Paradigms and Assumptions (1960s–1990s)

Early paradigms of depression research became largely organized around stress, monoamines, and the hypothalamic–pituitary–adrenal axis as the primary translators of adversity into “behavioral despair” [2,17]. Forced swim and learned helplessness tests, later complemented by chronic stress paradigms in rodents and primates, operationalized immobility, withdrawal, and subdued exploration as proxies for hopelessness [18,19]. These models were highly sensitive to monoaminergic and hypothalamic–pituitary–adrenal (HPA) axis-modulating drugs and, for a time, appeared to offer a direct bridge between synaptic mechanisms and clinical symptoms [20,21]. Parallel work on glucocorticoid feedback, neurosteroids, and early immune and gut–brain signals refined this picture, yet still treated depression largely as a unitary endpoint of a relatively linear stress–monoamine–hypothalamic–pituitary–adrenal (HPA) axis cascade [22,23].

On the clinical side, the introduction of DSM III and DSM III R reconfigured nosology around symptom checklists, while the Hamilton Depression Rating Scale (HAM-D), and later the Montgomery–Åsberg Depression Rating Scale (MADRS), scores became the dominant trial endpoints [24,25]. Response and remission were quantified by cutoffs on these scales, and short, fixed-duration trials with single primary mood outcomes became the gold standard for regulatory sensitive evidence [26,27]. Placebo trials in phases, designed to screen out early improvers and sharpen drug signal, further shaped effect sizes and sample composition, often at the cost of ecological validity [26,28]. Translation across trials relied on scale equivalence rather than deeper phenotyping or mechanistic anchors [29,30].

Within this framework, crucial sources of heterogeneity were marginalized [31]. Sex- and age-dependent profiles of mood, anxiety, and somatic symptoms were rarely modeled explicitly in either preclinical or clinical research, despite emerging data on sex-specific monoamine and HPA signatures [32,33,34,35]. Ancestry, social context, chronic pain, and subthreshold anxiety were treated as noise, not structure [36]. An overreliance on narrow, short-term symptom endpoints, focused on short-term mood changes in narrowly defined samples, left longer-term trajectories, functional outcomes, and comorbidity patterns largely uncharted, planting many of the seeds for the replication, generalization, and treatment-resistance challenges that later decades would confront [37,38,39].

## 3. Key Mechanistic Pivots in Systems Psychiatry

This section outlines ten mechanistic domains that collectively illustrate the transition from monoamine-centered models of depression to a multiscale systems psychiatry framework, with each subsection integrating a defined biological mechanism with its translational implications. Each section focuses on a specific mechanistic targe—plasticity and circuit dynamics, reward and stress alignment, immune–metabolic and genomic milieu, or multi-point intervention logic—and links it to concrete translational readouts. Together, these pivots outline how synapses, circuits, body-wide signals, and trial architectures can be jointly tuned to redesign treatment-resistant depression (TRD) as a tractable, stratified systems problem.

### 3.1. Plasticity and Circuit Control of Depressive States

Across these cards, depression science shifts from monoamine scarcity toward dynamic systems. Each mini-anchor foregrounds mechanisms, actionable readouts, and clinically tractable targets that scaffold next-generation translational trial designs.

#### 3.1.1. Synaptic Plasticity and Intrinsic Excitability

At the most proximal level, synaptic plasticity and intrinsic excitability reframe depression as a disorder of experience-dependent circuit wiring rather than as a simple transmitter shortage [40,41]. Long-term potentiation and depression, AMPA receptor throughput, and evoked excitatory postsynaptic potentials index rapid synaptic remodeling, while neurogenesis and ERK-sensitive priming, including ketamine-induced effects, extend plasticity across longer timescales [42,43,44,45]. Importantly, early electroencephalography, local field potentials, resting-state functional magnetic resonance imaging, and AMPA-forward assays provide convergent translational readouts that anchor plasticity as a measurable and clinically actionable target [46,47].

#### 3.1.2. Glutamate/γ–Aminobutyric Acid (GABA) Microcircuit Control

Building on synaptic plasticity, glutamate and γ-aminobutyric acid microcircuits define how excitation–inhibition balance is locally implemented within cortical networks. Somatostatin- and parvalbumin-expressing interneurons tune affective set points by shaping oscillatory dynamics and information flow across layers [48,49,50,51]. Within this framework, N-methyl-D-aspartate (NMDA) and metabotropic glutamate receptor modulation, inhibitory postsynaptic potentials, and layer-specific oscillations emerge as key mechanistic levers [52,53,54]. Translationally, oscillation-anchored endpoints, task-locked transcranial magnetic stimulation–electroencephalography, and interneuron-specific computational or animal models link microcircuit dysfunction to scalable EEG and behavioral signatures [55,56].

#### 3.1.3. Circuit-Level Nodes (Drugs and Devices)

At the network scale, microcircuit and plasticity deficits converge onto identifiable circuit-level nodes that are now directly targeted by drugs and devices [57,58]. Networks linking the habenula, subcallosal cingulate, and ventromedial prefrontal cortex serve as intervention points for deep brain stimulation, vagus nerve stimulation, and transcranial magnetic stimulation, including accelerated theta-burst protocols guided by connectivity-informed targeting and TMS-EEG physiology [59,60]. Importantly, proof of target engagement, optimized dosing schedules, and responder enrichment based on baseline network topology redefine how device parameters translate into durable clinical outcomes, moving neuromodulation toward precision deployment [61,62].

### 3.2. Reward, Motivation, and Stress Systems

Where plasticity and microcircuits define how affective states are encoded, reward and stress systems determine why motivation collapses and fatigue persists in many depressive phenotypes. Stress exposure reshapes reward valuation and effort allocation, biasing behavior toward withdrawal and anergia rather than adaptive pursuit. This pivot integrates mesolimbic reward processing with stress-responsive neuromodulatory and endocrine systems, positioning anhedonia and fatigue as system-level failures rather than isolated symptoms.

#### 3.2.1. Reward, Motivation, and Stress–Opioid Tone

At the core of motivational dysfunction, anhedonia reflects disrupted mesolimbic signaling in which dopamine-driven reward processing is gated by dynorphin-mediated κ-opioid stress tone [63,64]. Stress-induced activation of κ-opioid receptors suppresses phasic dopamine release, shifting behavior away from effortful reward-seeking and toward passive coping [65,66,67,68]. Translationally, effort-based choice tasks, ventral striatal activity, and inflammatory status provide convergent readouts of motivational collapse, while selective κ-opioid antagonists and κ-opioid-modulating antidepressants offer mechanistically targeted strategies to dissociate hedonic capacity from stress-induced motivational inhibition [69,70].

#### 3.2.2. HPA–Circadian–Stress Axis

Motivational and reward deficits are further shaped by coupling between the hypothalamic–pituitary–adrenal axis and circadian timekeeping systems. Glucocorticoid receptor sensitivity, cortisol rhythmicity, and sleep architecture jointly tune emotional regulation and energy allocation across the day [71,72,73,74]. Disrupted dim-light melatonin onset, altered actigraphy, and a dysregulated cortisol awakening response signal internal misalignment that amplifies fatigue, negative affect, and relapse vulnerability [75,76,77]. In this context, chronotype-aware protocols, sleep-linked endpoints, and glucocorticoid receptor assays operationalize stress–circadian dysfunction as a modifiable treatment dimension rather than background noise [78,79].

### 3.3. Immune–Metabolic–Genomic Modifiers of Risk and Treatment Response

If synaptic and circuit mechanisms define how depressive states are encoded and reward–stress systems explain their functional expression, immune, metabolic, and genomic modifiers determine the biological context in which these processes unfold. Peripheral inflammation, metabolic load, and transcriptional regulation quietly bias neural plasticity, reward sensitivity, and treatment durability, transforming background physiology into a primary determinant of symptom profile and therapeutic response. This pivot reframes systemic signals not as epiphenomena, but as modulators that shape both risk and responsiveness across depressive subtypes.

#### 3.3.1. Tryptophan (Trp)–Kynurenine (KYN) Steering

Immune activation redirects tryptophan metabolism toward the KYN pathway, coupling peripheral inflammatory states to central neurotransmission and plasticity disruption [80,81]. Indoleamine 2,3-dioxygenase (IDO) and tryptophan 2,3-dioxygenase (TDO) shift kynurenine balance toward neuroactive metabolites, altering glutamatergic tone, microglial activation, and stress sensitivity [77,78,79,80]. Clinically, C-reactive protein (CRP) levels, KYN/Trp ratios, and quinolinic (QA)-to-kynurenic acid (KYNA) balance align with anhedonia, cognitive slowing, and fatigue-dominant symptom clusters, supporting inflammation-stratified trial designs and mechanistically guided adjunctive interventions in treatment-resistant depression [81,82,83,84,85,86,87].

#### 3.3.2. Neuroimmune and Glia

Beyond metabolic steering, neuroimmune signaling positions microglia and astrocytes as active regulators of synaptic strength and network tone. Complement pathways, colony-stimulating factor 1 receptor, and purinergic P2X7 signaling sculpt synaptic pruning, excitability, and circuit stability under inflammatory conditions [82,83,84]. Translationally, positron emission tomography ligands, cerebrospinal cytokine panels, and electroencephalographic complexity measures provide convergent markers of central immune engagement [85,86,87,88]. Importantly, incorporating sex, baseline inflammatory state, and stimulation-induced immune shifts as design variables reframes immune modulation as a tractable dimension for precision psychiatry [89].

#### 3.3.3. Metabolic–Endocrine Crosstalk

Metabolic–endocrine factors further constrain neural resilience by coupling energy regulation to mood and cognition. Insulin resistance, adiposity, and glucagon-like peptide-1 (GLP-1) signaling alter brain insulin sensitivity, reward processing, and response to antidepressants and neuromodulation [90,91,92]. Metabolic syndrome and type 2 diabetes (T2D) thus identify patient subgroups in whom standard treatments show reduced efficacy and durability [93,94]. Pragmatic trial designs that embed metabolic stratification and functional endpoints reposition metabolic load as a modifiable determinant rather than a comorbidity to be controlled away [90,95].

#### 3.3.4. Epigenetic/Transcriptional Gating

At the slowest timescale, epigenetic and transcriptional mechanisms stabilize depressive states and constrain recovery trajectories. Histone deacetylases, bromodomain proteins, lysine-specific demethylase 1, and DNA methyltransferases regulate chromatin accessibility and gene expression programs linked to stress sensitivity and plasticity [96,97]. Cell-type-resolved ATAC and RNA sequencing, paired with peripheral chromatin markers, tracks medication- and neuromodulation-induced state transitions [98,99,100]. Incorporating these measures into longitudinal trials supports durability-focused designs that distinguish transient symptom relief from sustained circuit reconfiguration [97,99,100].

### 3.4. Multi-Point Precision Strategies and Emerging Targets

As plasticity, reward–stress dynamics, and immune–metabolic context converge, treatment-resistant depression emerges not as a failure of single targets, but as a systems-level mismatch between intervention and patient-specific biology. This perspective reframes precision psychiatry as the strategic coordination of multiple levers—pharmacological, neuromodulatory, psychotherapeutic, and digital—aligned with defined biotypes rather than syndromic averages.

#### Multi-Point Strategies and Next-Wave Targets

Multi-point strategies treat treatment-resistant depression as overlapping, partially independent biotypes that require coordinated intervention across synaptic, circuit, immune, and behavioral domains [101,102,103,104]. Biomarker-informed sequencing, pharmacogenomic guidance, and biotype-specific combinations replace linear, trial-and-error escalation with adaptive logic that exploits mechanistic complementarity [101,105,106,107]. Translationally, adaptive platform trials, N-of-1 designs, and digitally enriched endpoints allow for rapid iteration while preserving individual-level inference, shifting evaluation from short-term symptom reduction toward durability, functioning, and real-world effectiveness [102,103,104,107].

## 4. Divergence → Reconnection

As monoamine and stress-based models began to fray, a new translational strategy sought bridges that could genuinely scale across species rather than relying on face validity alone [108]. Electrophysiological motifs, such as frontal alpha asymmetry and reward related event-related potentials, emerged as candidate intermediate phenotypes that can be measured in rodents and humans using homologous paradigms [109,110,111]. Sleep architecture, particularly REM density and slow wave fragmentation, offered another shared metric, mirrored by alterations in stress-exposed animals and patients with melancholic or TRD [112,113,114]. In parallel, probabilistic reward tasks and cognitive control batteries were adapted across rodents, non-human primates, and humans, while longitudinal inflammatory and endocrine panels were integrated into both preclinical and clinical protocols, enabling richer cross-species modeling of reward inflammation coupling [115,116].

However, the field also accumulated clear evidence of repeated failure to incorporate lessons from earlier methodological missteps [117]. The assumption that immobility equals despair persisted long after it became evident that forced swim behavior reflects a narrow coping style, not the multidimensional construct of depression [118,119]. Short-duration trials and brief stress paradigms continued to dominate despite growing evidence that synaptic, circuit, and immune remodeling unfold over weeks to months [120,121]. Single-endpoint thinking, usually centered on global depression scores or single behavioral tests, repeatedly obscured domain-specific gains and masked heterogeneity in trajectories [122,123].

The most promising advances have come from deliberate back-translation [124,125,126]. Symptom clusters and rating scale factors are first decomposed into Research Domain Criteria (RdoC)-like domains of negative valence, reward processing, arousal, and cognition, and are then re-instantiated as tractable tasks and physiological readouts in animals [125,127]. Multimodal biomarker programs now use human-defined phenotypes to guide the design of cross-species EEG batteries, reward and cognitive paradigms, and peripheral immune signatures [128,129]. In doing so, they begin to reconnect bench and bedside around shared dimensions rather than diagnostic labels alone [130,131] (Table 1).

## 5. Clinical Applications Today

Clinical applications of systems psychiatry are increasingly shifting beyond one-size-fits-all algorithms toward stratified care grounded in measurable biological and behavioral dimensions [132]. Depression is increasingly parsed into high-inflammation, metabolically burdened, anhedonia-dominant, and disrupted sleep or circadian profiles, overlaid on the formal staging of treatment resistance [133,134]. Elevated CRP altered neutrophil to lymphocyte ratios, and immune metabolic gene signatures delineate patients whose symptoms cluster around anergia and anhedonia, while sleep fragmentation and circadian misalignment identify another modifiable axis that cuts across stages of non-response [135]. Parallel work on pharmacogenomics, circuit-based biotypes, and age-specific EEG signatures is entering specialist clinics and early-phase trials [13,136,137].

A clear distinction now separates tools that are clinically ready from those that are almost ready [138]. Structured TRD staging, esketamine, neuromodulation devices, and several pharmacogenomic panels have achieved regulatory approval and are supported by comparative effectiveness data [106,139,140]. By contrast, multiplex inflammatory and metabolic panels, task-based cognitive emotional biomarkers, and digital phenotyping batteries remain largely in the candidate domain, often validated in carefully selected cohorts but not yet embedded in stepped care pathways [129,141].

**Figure 2:**
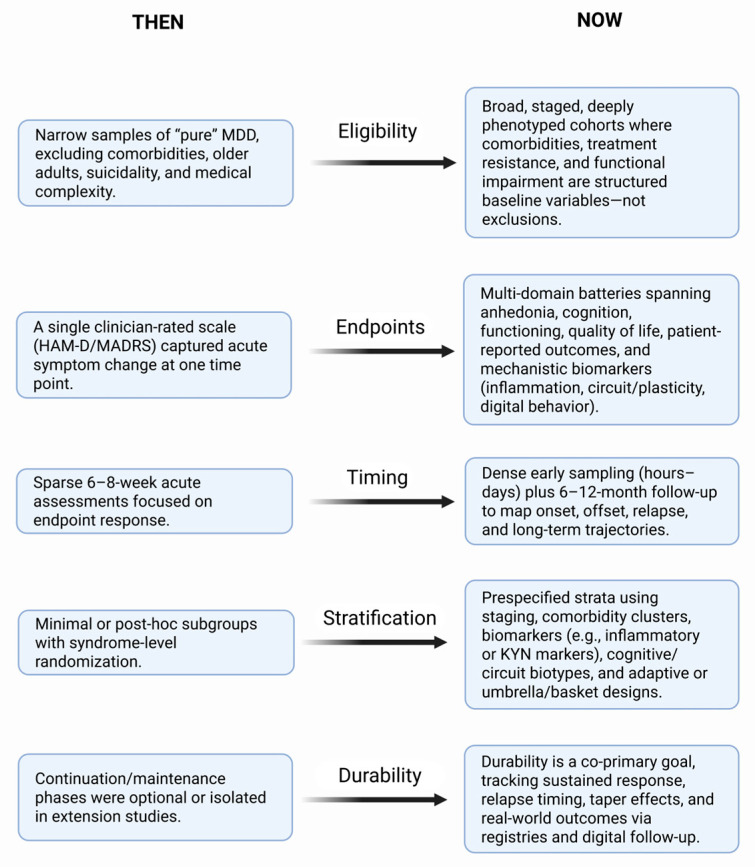
Evolution of depression trial design across eligibility, endpoints, timing, stratification and durability. The schematic contrasts conventional antidepressant trials (Then, **left**) with emerging systems-psychiatry-informed designs (Now, **right**). Eligibility. Earlier trials typically enrolled narrowly defined adults with “pure” major depressive disorder (MDD), excluding common comorbidities, older age, suicidality, and medical complexity; contemporary designs move toward staged, better-characterized, and more inclusive samples, with comorbidities, treatment resistance, and functional impairment captured as structured baseline variables rather than automatic exclusions. Endpoints. Traditional studies relied on a single clinician-rated symptom scale (for example, HAM-D or MADRS) at one acute time point; modern trials incorporate multi-domain outcome batteries, including anhedonia and cognition, functioning and quality of life, patient-reported outcomes, and mechanistic readouts (for example, inflammatory markers, circuit or plasticity measures, digital behavior). Timing. Classic trials emphasized 6–8-week acute response with relatively sparse visits; new designs layer dense early sampling windows (hours to days for rapid-acting agents) onto longer follow-up to 6–12 months, enabling characterization of onset, offset, and relapse dynamics. Stratification. Earlier studies used minimal or post hoc subgrouping (for example, melancholic versus atypical) and randomization only at the syndrome level; contemporary designs prespecify strata and enrichment rules based on clinical staging, comorbidity clusters, biomarkers (such as inflammation or KYN metrics), and cognitive or circuit biotypes, and sometimes deploy adaptive or umbrella/basket architectures. Durability. Historically, continuation and maintenance phases were optional or handled in separate extension studies; current trials increasingly embed durability as a co-primary objective, tracking time to relapse, sustained response, taper/discontinuation effects, and real-world effectiveness via registries or digital follow-up, thereby aligning trial design with the long-term course of depressive illness. Created in BioRender. Tanaka, M. (2025) https://BioRender.com/evdupmh (accessed on 1 December 2025). HAM-D, Hamilton Depression Rating Scale; KYN, kynurenine; MADRS, Montgomery–Åsberg Depression Rating Scale; MDD, major depressive disorder.

Translating these advances into everyday practice faces substantial hurdles [142]. Access to neuromodulation, ketamine-based interventions, and genomic testing is uneven, constrained by cost, infrastructure, and workforce training [143]. Regulatory frameworks still privilege drug over device and algorithm, and reimbursement rarely rewards stratified assessment [144]. Without deliberate attention to equity, biologically enriched care risks deepening disparities, as populations with higher inflammatory burden, multimorbidity, and limited digital access may be the least likely to receive precision-guided interventions [145,146,147] (Figure 2).

## 6. What We Got Wrong/Right

Many of the field’s “failures” now look like solvable design problems rather than fatal errors [148,149]. Trial-and-error prescribing, short-term symptom-focused trials, and inflated remission estimates exposed what happens when heterogeneity is ignored and protocols drift [148,150,151]. The corrective principles are clearer now: implement decision support and data-driven personalization, redesign trials to prioritize long-term functioning and real-world effectiveness, and adhere strictly to pre-specified protocols with transparent reporting [152,153,154]. The concept of difficult-to-treat depression and consensus definitions of TRD are direct products of these lessons [104,155,156].

A second cluster of mistakes involved what and whom we chose to measure [157,158]. Overbroad diagnostic criteria without biomarkers, unclear psychotherapy “active ingredients,” late recognition of comorbidity, and neglect of patient preference and digital engagement all narrowed impact [148,159,160,161]. The responses are already reshaping practice [162]. Biomarker-informed and endophenotype-based frameworks, component-focused psychotherapy trials, integrated care models, shared decision tools, guided digital and blended interventions, and Delphi-based TRD guidelines collectively represent durable gains and a more realistic architecture for precision psychiatry [104,156,160,163].

## 7. Outlook

The next decade will be judged by whether systems psychiatry can be changed from an attractive narrative into falsifiable science [164,165]. Two preclinical predictions are within reach. First, that manipulating specific prefrontal–striatal and prefrontal–limbic circuits in sex-balanced, chronic stress models will yield biotypes that map onto human imaging, defined network patterns, and differential treatment response [166,167,168]. Second, that patient-derived cellular systems combined with multi-omic profiling will prospectively predict which immune and metabolic perturbations in animals reproduce the anhedonia-dominant, high-inflammation phenotypes observed in humans [135,169].

On the clinical side, trial design must shift in two concrete ways. Large, multivariate, cross-trial prediction studies should be used not only to detect average effects but to learn stable individual-level treatment rules [170,171,172]. In parallel, factorial and adaptive designs need to dissect psychotherapy and combined “active ingredients” for treatment, rather than testing monolithic packages [173,174,175,176].

A viable implementation pathway is hybrid effectiveness implementation research embedded in integrated care, where stratification tools, targeted pharmacotherapy, and digital monitoring are co-designed with health systems [126,177]. Underpinning all of this should be a shared data standard that harmonizes symptom networks, EEG and imaging markers, and trial metadata, with preregistered analytic pipelines and mandatory external validation [178,179].

## 8. Conclusions

In this perspective review, I propose plasticity-centered systems psychiatry as the key lens through which both model-building and bedside decisions can be restructured. Synaptic remodeling, circuit dynamics, immune–metabolic context, and sleep-dependent homeostasis are repositioned as explicit, testable levers for intervention rather than diffuse background processes. A concrete practice change is to implement structured, plasticity-informed care pathways that link symptom profiles to simple circuit-sensitive indices such as reward tasks, sleep and circadian metrics, and low-burden immune or metabolic panels. A parallel research change is to develop adaptive, multimodal platforms in which the ten subsequent papers systematically interrogate promising synaptic, microcircuit, network, immune, and chronobiological targets for depression treatment.

## Figures and Tables

**Table 1:** Cross-species bridge map for depression-relevant constructs and translatable assays. Cross-species bridge map linking human constructs to preclinical assays, quantitative readouts, and clinical analogs that can be implemented in contemporary trials. “Status” summarizes translational maturity (routine, emerging, or exploratory), while “Design tip” highlights one concrete way to embed each bridge into mechanistically anchored, heterogeneity-aware study designs.

Human Construct	Preclinical Assay	Readout	Clinical Analog	Status	Design Tip
Anhedonia/motivational deficit	Effort-based decision tasks (progressive ratio, T-maze barrier, operant sucrose)	Breakpoint, lever presses, willingness to work under stress or inflammation	Probabilistic reward tasks, EEfRT, ventral striatal BOLD, anhedonia scales	Emerging trial biomarker	Separate hedonic “liking” from motivational “wanting”; include stress/inflammation challenge blocks.
Negative affect/threat bias	Fear conditioning and extinction; chronic social defeat	Freezing/avoidance, extinction curves, startle, social withdrawal	Fear-learning and extinction tasks, startle paradigms, threat-bias tasks in anxious/MDD subgroups	Robust basic science; limited clinical use	Use as domain-specific endpoint in anxious and trauma-loaded depression; pair behavior with EEG/fMRI.
Cognitive control/executive dysfunction	Attentional set-shifting, 5-CSRTT, reversal learning	Errors, omissions, reaction times, perseveration indexes	Set-shifting (e.g., CANTAB), n-back, Stroop, Trail Making, DLPFC activation	Secondary endpoint in several trials	Pre-stratify “cognitively loaded” depression; link change to functioning and return-to-work outcomes.
Sleep and circadian disruption	Rodent EEG/EMG with chronic stress or light-cycle shift; REM-deprivation models	REM latency/density, NREM slow-wave power, activity rhythms, phase shifts	Polysomnography, actigraphy, DLMO, sleep/circadian questionnaires	Strong observational; emerging endpoints	Align dosing and assessments with chronotype; treat sleep/circadian metrics as primary modifiable targets.
HPA axis and stress reactivity	Chronic mild stress, restraint, social defeat; Dex/CRH challenges	Corticosterone profiles, GR sensitivity, coping style, stress-induced behavioral shift	Cortisol awakening response, DST, lab stress tests, hair cortisol	Mixed but promising for subtyping	Sample across diurnal cycle; co-model stress markers with symptom domains (anergy, anxiety, cognitive fog).
Inflammation–KYN steering	LPS/IFN-α or stress-sensitized immune activation; Trp–KYN pathway assays	KYN/Trp ratio, QA/KYNA balance, microglial activation, cytokine panels	CRP, IL-6/TNF panels, plasma KYN/Trp, symptom clusters (anergia, anhedonia, psychomotor slowing)	High translational interest	Pre-specify “inflammation-high” strata; collect longitudinal KYN panels and align with treatment response.
Metabolic–endocrine load	High-fat diet, genetic obesity, insulin-resistance models	Glucose tolerance, insulin signaling, adiposity, spontaneous activity	BMI, waist-to-hip ratio, HOMA-IR, HbA1c, metabolic-syndrome indices	Growing but underused in trials	Embed metabolic panels into TRD studies; design dedicated obesity/T2D depression trials with functional endpoints.
Synaptic plasticity/rapid-acting response	Ketamine/psychedelic paradigms; LTP/LTD, in vivo spine imaging, AMPA-forward assays	Spine density, AMPA/NMDA ratio, LTP/LTD magnitude, early oscillatory changes	Early EEG/MEG plasticity markers, TMS-LTP readouts, 24–72 h symptom and cognition shifts	Strong mechanistic, clinical for ketamine	Build in early (24–72 h) windows and plasticity markers as key secondary endpoints in rapid-acting trials.
Network-level connectivity biotypes	Chemogenetic/optogenetic PFC–striatal/limbic manipulation; rodent rsfMRI/EEG	Resting-state connectivity, oscillatory coupling, causal node influence, behavior under circuit control	rsfMRI biotypes, TMS-EEG connectivity, SCC/vmPFC network markers for neuromodulation targeting	Emerging targeting tool	Require “target engagement” thresholds for drugs/devices; enrich samples by baseline network topology.
Digital behavior and passive monitoring	Home-cage automated monitoring of movement, sleep, and social interaction	Continuous activity, sleep–wake structure, social proximity, exploration patterns	Smartphone-based mobility, call/text patterns, speech and behavior passively captured by sensors	Early exploratory	Predefine digital endpoints (e.g., mobility, social withdrawal) and link them to functional and relapse outcomes.

5-CSRTT, five-choice serial reaction time task; BMI, body mass index; AMPA, α-amino-3-hydroxy-5-methyl-4-isoxazolepropionic acid; BOLD, blood oxygen-level-dependent; CANTAB, Cambridge Neuropsychological Test Automated Battery; Dex/CRH, dexamethasone/corticotropin-releasing hormone; DLPFC, dorsolateral prefrontal cortex; DLMO, dim light melatonin onset; DST, dexamethasone suppression test; EEG, electroencephalograph; EMG, electromyography; fMRI, functional magnetic resonance imaging; GR, HbA1c, glycated hemoglobin; glucocorticoid receptor; HOMA-IR, homeostatic model assessment of insulin resistance; HPA, hypothalamic–pituitary–adrenal; IFN-α interferon-alpha; IL-6, interleukin-6; KYN, kynurenine; LTD, long-term depression; LPS, lipopolysaccharide; LTP, long-term potentiation; MEG, magnetoencephalography; NMDA, N-methyl-D-aspartate; NREM, non-rapid eye movement; PFC, prefrontal cortex; QA, quinolinic acid; REM, rapid eye movement; rsfMRI, resting-state functional magnetic resonance imaging; SCC, subcallosal cingulate; T2D, type 2 diabetes; TMS, transcranial magnetic stimulation; TNF, tumor necrosis factor; TRD, treatment-resistant depression; Trp, tryptophan; vmPFC, ventromedial prefrontal cortex.

## Data Availability

No new data were created or analyzed in this study. Data sharing is not applicable to this article.

## Abbreviations

The following abbreviations are used in this manuscript:

5-CSRTT	five-choice serial reaction time task
AhR	aryl hydrocarbon receptor
AMPA	α-amino-3-hydroxy-5-methyl-4-isoxazolepropionic acid
ATAC	assay for transposase-accessible chromatin
BMI	body mass index
BOLD	blood-oxygen-level–dependent
CANTAB	Cambridge Neuropsychological Test Automated Battery
CRP	C-reactive protein
CSF1R	colony-stimulating factor 1 receptor
Dex/CRH	dexamethasone/corticotropin-releasing hormone
DLMO	dim light melatonin onset
DLPFC	dorsolateral prefrontal cortex
DNMTs	DNA methyltransferases
DSM	Diagnostic and Statistical Manual of Mental Disorders
EEG	electroencephalography
EefRT	Effort Expenditure for Rewards Task
eEPSPs	evoked excitatory postsynaptic potentials
ERK	extracellular signal-regulated kinase
EMG	electromyography
fMRI	functional magnetic resonance imaging
GABA	γ-aminobutyric acid
GLP-1	glucagon-like peptide-1
GR	glucocorticoid receptor
HAM-D	Hamilton Depression Rating Scale
HbA1c	glycated hemoglobin
HDACs	histone deacetylases
HOMA-IR	homeostatic model assessment of insulin resistance
HPA	hypothalamic–pituitary–adrenal axis
IDO	indoleamine 2,3-dioxygenase
IFN-α	interferon-alpha
IL-6	interleukin-6
IPSPs	inhibitory postsynaptic potentials
κ	kappa
KYN	kynurenine
KYNA	kynurenic acid
LFP	local field potential
LPS	lipopolysaccharide
LSD1	lysine-specific demethylase 1
LTD	long-term depression
LTP	long-term potentiation
MADRS	Montgomery–Åsberg Depression Rating Scale
MDD	major depressive disorder
MEG	magnetoencephalography
NMDA	N-methyl-D-aspartate
PET	positron emission tomography
PFC	prefrontal cortex
QA	quinolinic acid
RDoC	Research Domain Criteria
REM	rapid eye movement
RNA	ribonucleic acid
rsfMRI	resting-state functional magnetic resonance imaging
SCC	subcallosal cingulate cortex
T2D	type 2 diabetes
TDO	tryptophan 2,3-dioxygenase
TMS	transcranial magnetic stimulation
TMS-EEG	transcranial magnetic stimulation–electroencephalography
TNF	tumor necrosis factor
TRD	treatment-resistant depression
Trp	tryptophan
vmPFC	ventromedial prefrontal cortex
VNS	vagus nerve stimulation
